# Yellow Fever in Philadelphia

**Published:** 1853-10

**Authors:** 


YELLOW FEVER IN PHILADELPHIA.

A few cases of yellow fever have occurred in Philadelphia, under circumstances that strongly favor the theory of its portability. It seems clear that the pestilence was carried there by a ship. No apprehension is entertained that the disease will become epidemic in that city. The *Philadelphia Sun*, in recording the fact that the Board of Healt reports two fatal cases of the fever in a week, remarks that the disease exists still in the neighborhood of South and Swanson streets, to which locality it was brought by the barque *Mandarin;* but it is of a mild type, and, in most instances, yields to medical treatment. Many houses in the vicinity have been deserted, and several factories closed. The disease, however, it is said, is confined almost exclusively to badly ventilated localities, to purify which the Board of Health is doing every thing in its power, but it is supposed it will require a few heavy frosts to kill it off effectually.

